# Effects of Dietary Tuber Ethanolic Extract of Nut Grass (*Cyperus rotundus* Linn.) on Growth, Immune Response, and Disease Resistance in Nile Tilapia (*Oreochromis niloticus*)

**DOI:** 10.3390/ani14030503

**Published:** 2024-02-02

**Authors:** Supranee Wigraiboon, Ruamruedee Panchan, Vijitra Luang-In, Wilailak Ounjit, Paiboon Panase, Sontaya Sookying, Nantaporn Sutthi

**Affiliations:** 1 Department of Agricultural Technology, Faculty of Technology, Mahasarakham University, Maha Sarakham 44150, Thailand; supranee.w@msu.ac.th (S.W.); ruamruedee.p@msu.ac.th (R.P.); 2 Applied Animal and Aquatic Sciences Research Unit, Division of Fisheries, Faculty of Technology, Mahasarakham University, Maha Sarakham 44150, Thailand; 3 Natural Antioxidant Innovation Research Unit, Department of Biotechnology, Faculty of Technology, Mahasarakham University, Maha Sarakham 44150, Thailand; vijitra.l@msu.ac.th; 4 Department of Sociology and Anthropology, Faculty of Humanities and Social Sciences, Mahasarakham University, Maha Sarakham 44150, Thailand; wilailak.o@msu.ac.th; 5 Fisheries Division, School of Agriculture and Natural Resources, University of Phayao, Phayao 56000, Thailand; tong33_panamagigas@hotmail.com; 6 Unit of Excellence Physiology and Sustainable Production of Terrestrial and Aquatic Animals (FF66-UoE014), School of Agriculture and Natural Resources, University of Phayao, Phayao 56000, Thailand; sontaya.so@up.ac.th; 7 Division of Pharmaceutical Sciences, School of Pharmaceutical Sciences, University of Phayao, Phayao 56000, Thailand

**Keywords:** *Cyperus rotundus*, growth performance, immune response, disease resistance, Nile tilapia

## Abstract

**Simple Summary:**

This study investigates the use of tuber extract from nut grass (*Cyperus rotundus* Linn.) as growth and immune boosters in aquaculture. Although *C. rotundus* is considered a weed of low value, it has been found to enhance growth and act as an immunostimulant in Nile tilapia. *C. rotundus* exhibited antimicrobial activity against *S. agalactiae*, resulting in increased fish survival. The study suggests that the immunostimulant properties of *C. rotundus*, typically regarded as a weed of low value, could serve as a value-added source in a bio-green aquaculture model, promoting fish-friendly practices for consumer health.

**Abstract:**

Nut grass (*Cyperus rotundus* Linn.) is a weed that grows in all tropical, subtropical and temperate regions of the world, including areas where it grows on saline soil. This research was conducted to evaluate the effect of *C. rotundus* tuber extract in the diet on the growth performance and disease resistance of Nile tilapia. Various components of phytochemical importance of nut grass, including sugars/carbohydrates, terpenoids, tannins, and flavonoids were found in *C. rotundus*. Tilapia (*n* = 25 fish/group in triplicate) were fed with different levels of nut grass extract including 0 (control; T1), 0.4 (T2), 0.8 (T3), and 1.6 (T4) g/kg for 60 days in a completely randomized design (CRD) experiment. After the feeding trial, the highest weight gain and average daily gain (ADG) were observed in the T4 group, but it was not significantly different from T3 (Nile tilapia fed with a 0.8 g/kg) (*p* > 0.05). The lowest feed conversion ratio (FCR) was observed in the T3 group. Moreover, the fillet, crud lipid content, and blood chemical profiles (aspartate aminotransferase (AST), cholesterol, and malondialdehyde (MDA)) in fish fed with 1.6 g/kg were highest when compared in all groups. In addition, the T3 group presented with the immune response parameter found in red blood cells (RBC), lysozyme activity, and antioxidant (superoxide dismutase activity (SOD)) being higher than those of the control group (*p* < 0.05). The highest survival (93.33%) was observed in fish fed with 0.8 g/kg (T3) after a 14 day challenge with *Streptococcus agalactiae*. Thus, it was concluded that nut grass extract at 0.8 g/kg can be used to improve the growth performance and the tendency for resistance to *S. agalactiae* in Nile tilapia.

## 1. Introduction

Aquaculture production across the world has shown a continuous increase associated with high consumer demands including the dwindling of global capture fisheries. Concerning fisheries output, Thailand was ranked among the top 25 countries in 2023. *Oreochromis niloticus*, commonly referred to as Nile tilapia, holds significance in aquaculture due to its rapid growth, good survival and reproductive capabilities in captivity, high-quality meat, and favorable market value [1]. It is adaptable to a wide range of environmental conditions such as water temperature (8–42 °C) [2,3,4,5] and fresh to brackish water (0–20 ppt. salinity level) [6,7,8]. As of 2021, global production of this fish exceeded 4,827,581 tons across more than 120 countries [1]. Nile tilapia is economically importance as a food fish for many countries and is ranked as the world’s third most produced fish species after grass carp (*Ctenopharyngodon idellus*) and silver carp (*Hypophthalmichthys molitrix*) [1,9]. As the development of Nile tilapia has increased over the last few decades, the fish are mostly farmed using floating net cages, tanks, or earth ponds with a high fish stocking density. They are fed with high protein levels and water quality is monitored to obtain the highest production in a short period of time [10,11]. However, during intensive culture, the use of inappropriate rearing conditions and rapid changes in water qualities expose the fish to stress with many subsequent problems such as disease outbreak [12,13]. Fish farmers often encounter disease infection caused by bacteria, viruses, and parasites during farming, leading to a significant economic loss and subsequent loss globally, with extensive waste for the fish culture industry [14,15,16,17,18,19,20]. Among such disease infections, *Streptococcus agalactiae* (or group B streptococcus; GBS) is the one of the main causes of considerable morbidity and mortality in cultured tilapia worldwide [21,22,23]. 

To treat *streptococcus* outbreaks, Nile tilapia vaccines have been recently developed [24,25]. Antibiotics are also applied (lincomycin, norfloxacin, oxytetracycline, ampicillin, erythromycin and chloramphenicol, oxolinic acid, gentamicin, sulfamethoxazole, and trimethoprim [26]). However, antibiotic resistance and residues in meat are recent concerns, particularly for export producers. Many herbs are promoted as alternative plant medicinals to treat disease outbreaks and reduce antibiotic usage. If these herbaceous plant organs are effective, they may be fish-friendly and beneficial to consumer health [27,28].

Nut grass (*Cyperus rotundus*), belonging to the family Cyperaceae (sedges), is a weed with an erect triangular stem branching into three stems of purple, antenna-like seedpods. It is widely distributed in tropical, subtropical, and temperate regions throughout the world [29]. This plant is among the world’s most invasive species, absorbing nutrients from the soil and thereby reducing agronomic and horticultural crop yields [30,31,32,33]. However, the plant is traditionally used as a folk medicinal herb for the treatment of many diseases in humans, such as diarrhea, diabetes, pyresis, inflammation, malaria, and stomach and bowel disorders [34,35,36,37,38] due to the plant containing highly active ingredients in the form of essential oils, phenolic acids, tannins, glycosides, ascorbic acids, furochromones, monoterpenes, sesquiterpenes, sitosterol, alkaloids saponins, terpenoids, and flavonoids in the tuber and rhizomes [29,39,40,41,42]. In a few reports, nut grass (*C. rotundus*) has been employed in aquaculture. For instance, Citarasu et al. (2015) [43] recorded that extracts from *C. rotundus* effectively suppressed the white spot syndrome virus (WSSV) and enhanced the immune system in Indian white shrimp (*Fenneropenaeus indicus*), providing protection against WSSV infection. Additionally, Guo et al. (2019) [44] demonstrated the antibacterial activity of an aqueous extract of *C. rotundus* against *Streptococcus agalactiae*. The supplementation of *C. rotundus* tuber extract was observed to enhance feed palatability and stimulate the growth of *Cirrhinus mrigala* [45]. Nevertheless, there has been little research on the impact of extracted *C. rotundus,* as a supplementary feed, on fish growth, particularly in terms of immune responses against *S. agalactiae* in Nile tilapia. The aim of this study is to assess the impact of diet supplementation with the extract of *C. rotundus* on the growth performance, hematological parameters, and immune response of Nile tilapia against *S. agalactiae*. The hypothesis of this study is that the utilization of nut grass (*C. rotundus*) as an effective immunomodulator could enable the reduction of antibiotic usage in aquaculture, promoting fish-friendly practices for consumer health.

## 2. Materials and Methods

### 2.1. Preparation Ethanolic Extract from Tuber C. rotundus

Pure nut grass tuber (*C. rotundus*) dry powder was obtained from Thaprachan Herb Co., Ltd. (Bangkok, Thailand). Fresh *C. rotundus* tubers (size ranged 0.5–1.0 cm; aged 9–10 months) were collected from the wild at a location in Surin Province, Thailand. It was cleaned and sliced into small segments and subjected to an air-drying procedure. Subsequently, the dried pieces were mashed into a powder, which was then stored at a temperature of 4 °C until use. The tuber extraction procedure followed our previous report [46] with a slight modification. Briefly, the *C. rotundus* powder was combined with a 95% ethanol solution in a weight-to-volume ratio of 1:2, within 1000 mL conical flasks that were securely covered with aluminum foil. Flask contents were then macerated for 2 days at ambient temperature without agitation. After macerating, each mixture was filtrated through a sterile muslin cloth and Whatman qualitative filter paper. The resulting liquid residue was collected and dried using a rotary evaporator maintained at a temperature of 65 °C, and the resulting extract was kept at −20 °C until further analysis.

### 2.2. Phytochemical Screening

The standard procedures for *C. rotundus* phytochemical screening, as detailed in the protocol in [47,48], were used in the current study. Alkaloid presence was assessed through Dragendorff and Hager’s tests. The identification of cardiac glycosides, steroids, and terpenoids utilized the Liebermann-Burchard’s test. Molisch, Keller-Killiani, and Kedde’s tests were used to ascertain normal sugar, deoxy sugar, and the presence of an unsaturated lactone ring, respectively. For detecting cyanogenic glycosides, the sodium picrate method was applied. The frothing test was executed to determine the existence of saponins. In the case of screening for tannins, the lead acetate method was used to identify tannins and pseudotannins. Shinoda’s test was implemented for assessing the presence of flavonoids and Borntrager’s method was utilized to detect anthraquinones. The comprehensive protocol in these screenings follows our previously reported methodology [46].

### 2.3. Evaluation of Antioxidant Activity and Bioactive Compounds

The evaluation of the antioxidant activity of *C. rotundus* was carried out according to the methodology outlined [49]. The assessment encompassed the 2,2-diphenyl-1-picrylhydrazyl (DPPH) radical scavenging method, as well as the ferric reducing antioxidant power (FRAP) assay. The resultant values were expressed in terms mg Trolox equivalent (TE)/g DW for DPPH scavenging activity and mg Fe^2+^/g DW for FRAP activity. Furthermore, the bioactive constituents present in *C. rotundus* were analyzed for total phenolic content (TPC) and total flavonoid content (TFC), employing the method detailed in a previous study [50]. The quantified values were reported in mg gallic acid equivalent (GAE)/g DW for TPC, and mg rutin equivalent (RE)/g DW for TFC.

### 2.4. Fish and Experimental Diet

Sex reversed Nile tilapia (*Oreochromis niloticus*) fingerlings were bred at and purchased from Maha Sarakham Inland Fisheries Research and Development Center (Maha Sarakham, Thailand). The fish were placed in concrete ponds (size: 10 × 5 × 1.2 m) and allowed to acclimate for two weeks with a commercial diet containing 32% protein (Betagro^®^, Bangkok, Thailand). Afterward, 300 apparently healthy fish (weight 11.45 ± 1.41 g and length 6.85 ± 0.31 cm) were randomly distributed into 12 net cages (1 × 1 × 1 m) in concrete ponds, where the photoperiod was 12 h of light and 12 h of darkness throughout the experimental period. A completely randomized design (CRD) was used in this experiment. The fish was randomly divided into four groups (*n* = 25 fish/net cage) in triplicate experiments (T1: fish given only a basal diet, T2: given a *C. rotundus* extract 0.4 g/kg diet, T3: given a *C. rotundus* extract 0.8 g/kg diet, T4: given a *C. rotundus* extract 1.6 g/kg diet). 

For the experimental diets, the basal diet (commercial fish feed, Betagro^®^, Thailand) contained protein 32%, lipid 4%, moisture 12%, and fiber 6%. The ingredients of the commercial fish feed included fish meal, soybean meal, corn, broken rice, rice bran, as well as vitamins and minerals. The *C. rotundus* ethanol extract was mixed with the basal diet in four different proportions: 0, 0.4, 0.8 and 1.6 g/kg diets. In brief, crude *C. rotundus* extract was dissolved in 100 mL of 95% ethanol solution per kg diet, then sprayed into the experimental diet at different concentrations of 0.4, 0.8, and 1.6 g/kg per diet, respectively, and dried at room temperature. The feed pellets from all 4 groups were mixed with 20 g of guar gum (pellet binder), then coated with 4% agar solution at 20 mL/kg of the diet and air dried at 40 °C for 24 h. For the basal diet, added guar gum was also added with a coating of 4% agar solution but without the *C. rotundus* extract supplement. Diets were packed and stored at −20 °C until used, and this method was performed every 14 days. Proximate analysis data of the diets was obtained using standard methods [51], the results are shown in Table 1. Fish were manually fed with the experimental diets at a level of 5% of their respective body weight in two equal parts [28] for a period of 60 days. During the feeding trial, water was changed every two weeks and average water quality was monitored periodically, water temperature was recorded as 27.72 ± 0.47 °C, dissolved oxygen was 10.52 ± 0.73 mg/L, and pH was 8.69 ± 0.25.

### 2.5. Growth Measurements and Sample Collection 

The growth rate of all fish was determined using the mathematical growth model of Bagenal (1978) [52] to determine the weight gain (WG; g), average daily gain (ADG; g/day, specific growth rate (SGR; %/day), feed conversion ratio (FCR) and survival rate (%).

Weight gain (WG; g) = final weight (g) − initial weight (g);

Average daily gain (ADG; g/day) = [final weight (g) − initial weight (g)]/days;

Specific growth rate (SGR; %/day) = 100 × [{Ln final weight (g) − Ln initial weight (g)}/days];

Feed conversion ratio (FCR) = total feed (g)/weight gain (g);

Survival Rate (SR, %) = [number of survived fish/initial number of fish] × 100.

After 60 days of administering the experimental diets, blood was collected following our previous protocol [53]. Briefly, fish were anesthetized with 100 mg/L of clove oil and caudal vein blood samples were collected immediately. Blood (0.5 mL) was collected in and transferred to anticoagulant containers for hematological analysis. Another 1 mL of blood was deposited in sterile Eppendorf containers without anticoagulant in order to collect serum. Serum samples were kept at −20 °C until further analysis.

### 2.6. Body Chemical Composition and Organosomatic Indices 

Fillets of six fish/treatments were used for proximate body chemical composition using standard methods [51]. The organosomatic indices (%fillet, %carcass, hepatosomatic index (%HSI) and viscerosomatic index (%VSI)) were computed following the methods of Van Doan et al. (2023) [53]. 

### 2.7. Blood Chemical and Hematological Analysis 

The collected blood and serum samples were transported to the Veterinary Central Laboratory (Khon Kaen, Thailand) for comprehensive analysis of blood chemistry and blood indices parameters. These included measurements of serum aspartate aminotransferase (AST), alanine aminotransferase (ALT), blood urea nitrogen (BUN), total cholesterol (TC), total protein (TP), albumin (ALB), and globulin (GLB). Evaluation of hematological parameters involved the assessment of red blood cell (RBC), white blood cell (WBC), neutrophils (Neut), lymphocytes (Lymphs), hematocrit (Hct), and hemoglobin (Hb), following the detailed methodology outlined [53]. Furthermore, blood indices, such as mean cell volume (MCV), mean cell hemoglobin (MCH), and mean corpuscular hemoglobin concentration (MCHC), were determined according to the methodology established in [54]. 

### 2.8. Lipid Peroxidation and Activity of Antioxidant Enzymes 

The concentration of serum malondialdehyde (MDA) was determined by assessing thiobarbituric acid reactive substances (TBARS) using the method suggested in [55], with minor modifications as detailed in our previous report [56]. Catalase activity (CAT) in the liver was determined following the protocol outlined in [54]. The change in absorbance was measured after 20 s (A1) and 80 s (A2) of incubation at 240 nm and room temperature. The CAT value was calculated using the formula (A1 − A2)/0.0008. Superoxide dismutase (SOD) activity in the liver was measured and calculated according to the formulas provided in [54]. The percent of inhibition (%) = 100 − ((ΔA control − ΔA sample/ΔA control) × 100).

SOD activity (U/g liver) = % inhibition × 3.75.

### 2.9. Immunological Analysis

Lysozyme activity in serum samples was determined using the turbidimetric method, based on the lysis of the lysozyme-sensitive Gram-positive bacterium *Micrococcus lysodeikticus* (Sigma, Missouri, USA), as described [57] with some modifications as previously reported in [53]. The activity of myeloperoxidase (MPO) in serum was measured following the method suggested in [58], with slight modifications as detailed in [54]. And the serum bactericidal activity against *Streptococcus agalactiae* (10^3^ CFU/mL) was estimated using viable counts, in accordance with our previously established method [53].

### 2.10. Challenge Test 

After the feeding trial for 60 days, different sets of healthy fish (*n* = 10 fish/group in triplicate) were transferred and intraperitoneally challenged with a 0.3 mL suspension containing *Streptococcus agalactiae* at a dose of 10^8^ CFU/mL in 1× phosphate-buffered saline (PBS), following the method outlined in our previous study [53]. The negative control group was inoculated with 0.3 mL of 1× PBS. Following a post-challenge period of 14 days, the percentage of survival (%) of tilapia was observed, and the relative percentage of survival (RPS) was determined using the equation provided [59].

RPS = 1 − (% test mortality/% control mortality) × 100.

### 2.11. Statistical Analysis 

The acquired data were analyzed using a one-way analysis of variance (ANOVA), followed by Duncan’s post hoc test for multiple comparisons among the treatment groups. The significance level was set at *p* < 0.05. Results are presented as mean ± standard deviation (SD). 

## 3. Results

### 3.1. Phytochemical Screening, Antioxidant Activity and Bioactive Compounds 

The results for screened phytochemical constituents are shown in Table 2. It was found that sugars/carbohydrates, terpenoids, tannins, and flavonoids were present in the ethanolic extract of *C. rotundus* but alkaloids, anthraquinones, coumarins, saponins, steroids, cardiac glycosides, and cyanogenic glycosices were not. Antioxidant activity and bioactive contents were determined in *C. rotundus* as shown in Table 3. The results showed that *C. rotundus* had antioxidant activity such as DPPH scavenging activity and FRAP activity at 4.33 ± 0.04 mg TE/g of extract and 4.94 ± 0.05 mg Fe^2+^/g of extract, respectively. Moreover, the bioactive contents, total phenolic content (TPC) was 7.98 ± 0.17 mg GAE/g of extract, and the total flavonoid content (TFC) was concentration at 9.15 ± 0.81 mg RE/g of extract. 

### 3.2. Growth Performances

The effects of supplementing *C. rotundus* ethanolic extract in feed for 60 days on the growth performances of Nile tilapia are presented in Table 4. Fish fed with *C. rotundus* at a concentration of 1.6 g/kg of the diet (T4) demonstrated significantly improved growth performance (*p* < 0.05), particularly in terms of WG and ADG, when compared to the T2 and control group. The most favorable FCR was observed in fish fed the T3 diet. However, no significant differences were observed in SGR and SR among fish groups fed with *C. rotundus* and those on the control diet for all treatments (*p* > 0.05). 

### 3.3. Blood Chemical and Hematological Profiles 

The blood chemical profiles in this study are shown in Table 5. The activity of AST and TC were the highest in the T4 group, and significant differences were observed when compared to all other groups (*p* < 0.05). However, no significant differences (*p* > 0.05) were noticed in TP, GLB, ALT and BUN among all treatment groups. Hematological indices were assessed at the conclusion of the experiment and are presented in Table 6. The RBC count was notably lower in the T4 group in comparison to the T3 group (*p* < 0.05), although no significant differences were observed in comparison to the T2 and control groups (*p* > 0.05). Additionally, no significant differences were identified among all treatments (*p* > 0.05) with respect to WBC, Hb, Hct, Neut, Lymphs, MCV, MCH, and MCHC.

### 3.4. Body Composition and Organosomatic Indices 

The body composition and organosomatic indices were investigated at the end of the experiment and are presented in Table 7. There was a significant difference (*p* < 0.05) in the values for the fillet values, where fish fed with a *C. rotundus* supplement in their diet (T4) exhibited higher values compared to fish fed the T2, T3, and control diets. However, no significant differences were observed in the crude protein, crude lipid, moisture, ash, and carcass composition among the various experimental treatments (*p* > 0.05). Likewise, in terms of the organosomatic indices (HSI, VSI and SSI), the results indicated no significant differences (*p* > 0.05).

### 3.5. Immunological and Antioxidant Observations

The serum immunological and antioxidant activity observations of Nile tilapia receiving *C. rotundus* supplement in their diet were investigated at the end of the experiment and are presented in Table 8. A significant difference (*p* < 0.05) was observed in the content of serum LZM of fish fed the T3 diet, which was significantly higher than with the control diet (T1). However, for serum MPO, the results indicated no significant differences (*p* > 0.05) among the various treatments. In terms of antioxidant activity, liver SOD exhibited the highest values in fish fed the T3 diet, which was significantly higher than with the control diet (T1). Similarly, both the T3 and T2 groups demonstrated significantly greater CAT compared to the T4 group (*p* < 0.05). The MDA showed significant differences (*p* > 0.05) observed in the T4 group when compared to fish fed the T2, T3, and control diets (Table 8).

### 3.6. Bactericidal Activity and Challenge Test

**Figure 1:**
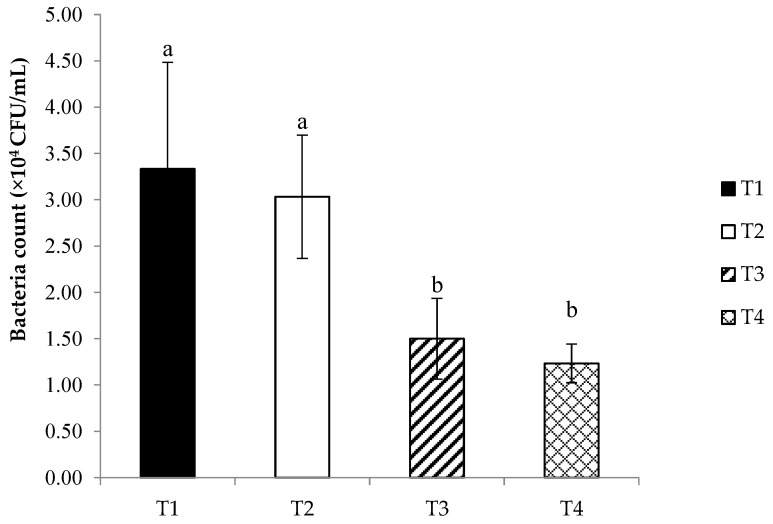
*Streptococcus agalactiae* counts after incubation with serum of Nile tilapia fed different concentrations of *C. rotundus*: T1 (0—control), T2 (0.4 g/kg diet), T3 (0.8 g/kg diet), and T4 (1.6 g/kg diet) for 60 days. Data are given as mean ± SD and different superscripts are significant differences (*p* < 0.05).

**Figure 2:**
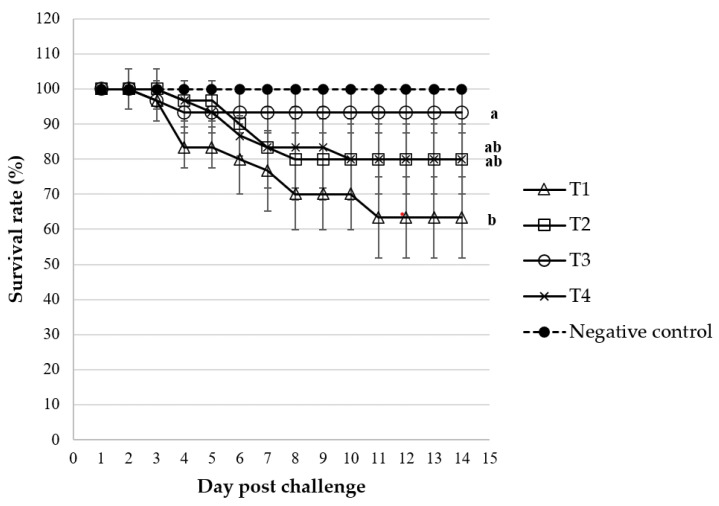
Percentage of survival rate of Nile tilapia fed different concentrations of *C. rotundus*: T1 (0.0—control), T2 (0.4 g/kg diet), T3 (0.8 g/kg diet), T4 (1.6 g/kg diet), and negative control (injected with PBS) for 14 days post the challenge test with *Streptococcus agalactiae*. Data are given as mean ± SD and different superscripts are significant differences (*p* < 0.05).

The results of the bacterial count of *Streptococcus agalactiae* after incubation with fish serum are presented in Figure 1. The results indicated that the lowest bacterial counts were obtained in fish fed the T4 diet, in comparison to the T2 and T1 diets (*p* < 0.05). However, no significant difference in bacterial counts between the T4 and T3 diets was observed. In the challenge test, the study revealed a significant improvement in the survival rate of Nile tilapia following the challenge with *S. agalactiae*, as depicted in Figure 2. This improvement was particularly pronounced in the group that received *C. rotundus* at a dietary concentration of 0.8 g/kg (T3), when compared to the T2, T4, and T1 control groups. Mortality within the control group was evident as early as day 3 post bacterial injection, while the *C. rotundus*-treated groups experienced fish mortality on days 4 to 5 following the *S. agalactiae* challenge. Notably, the relative percent of survival (RPS%) reached its highest value within the T3 diet-fed group (81.81%), followed by the T4 diet-fed group (45.45%) and the T2 diet-fed group (45.45%).

## 4. Discussion

The persistent issue of environmental contamination and the impact of chemical pollutants on aquatic organisms is of growing concern. Consequently, there is an increasing interest in exploring herbal remedies as alternatives for managing disease outbreaks, with a preference for these over antibiotics. To the best of our knowledge, nut grass (*C. rotundus*) serves as the focus of this study. Here, we embark on transforming *C. rotundus*, initially considered a low-value weed, into a high-value product. The ethanolic extract of *C. rotundus* showed significant improvements in parameters such as WG and ADG, along with an increase in the fillet percentage in Nile tilapia over a 60-day feeding period. The results of our study suggest that a *C. rotundus* extract dosage of 1.6 g/kg in the diet is suitable and can serve as an effective natural aquafeed additive to enhance growth in practical aquaculture. To date, there have been no reported investigations into the potential effects of *C. rotundus* on the growth of Tilapia species. This study represents the first exploration of *C. rotundus* as a dietary herbal supplement for fish, with a specific focus on Nile tilapia. Our findings align with the broader research in this domain. Notably, *Cyperus* powder, derived from this plant, is frequently employed as a cost-effective substitute for conventional feed components such as wheat bran or maize meal in fish diets. For instance, a study conducted by Rambabu et al. (2004) [45] demonstrated the successful replacement of wheat bran with *C. rotundus* powder, resulting in a significant increase in the WG of *C. mrigala* after a 45-day feeding period. Similarly, research by Oladele et al. (2010) [60] showed that substituting maize meal with Tigernut (*Cyperus esculentus*) at a 100% level led to substantial improvements in parameters such as WG, SGR, and overall catfish (*Clarias gariepinus*) production after an 8-week feeding period. The application of medicinal plants as growth enhancers, aimed at modulating physiological processes, is subject to a complex interplay of endogenous and exogenous factors. Within this context, the central nervous system (CNS) assumes a pivotal role in sensing and integrating these signals, and the resultant responses can significantly influence various aspects of development, growth, metabolism, and appetite [61]. Additionally, plant-derived compounds hold the potential to stimulate fish growth by modulating the somatotropic axis (growth hormones (GH), insulin-like growth factors (IGF-I and IGF-II)) and neuroendocrine pathways (ghrelin and leptin) [61]. However, further investigation is needed to elucidate the regulatory mechanisms involving *C. rotundus* in this context.

In line with these above findings, our study demonstrates that the ethanolic extract of *C. rotundus* tubers significantly promotes tilapia growth, possibly due to the presence of phytochemical compounds and bioactive elements within the plant. The screening of the ethanolic extract revealed the presence of four phytochemical elements: sugars/carbohydrates, terpenoids, tannins, and flavonoids. It is well-documented in earlier research that the extract of *C. rotundus* is rich in bioactive constituents, and the results of this study align with previous investigations [29,39,40,41,42]. In accordance with our findings, the phytochemical composition revealed in our data aligns with the conclusions of Babiaka et al. (2021) [62], who conducted an extensive review. They identified terpenoids and flavonoids as the primary bioactive constituents within *C. rotundus*, especially in plant samples primarily harvested from regions in Asia and Africa.

Furthermore, in our findings, we observed that *C. rotundus* exhibited antioxidant and bioactive compounds, as evidenced by its DPPH scavenging activity, FRAP activity, TPC, and TFC, as presented in Table 2. These results unequivocally establish the antioxidative potential of *C. rotundus*, consistent with previous reports [63,64]. Phenolic compounds hold a significant place in the composition of plants due to their antioxidant effects via deactivating lipid free radicals or inhibiting the breakdown of hydroperoxides into free radicals [65]. In fact, flavonoids have been documented to possess various beneficial properties, including antioxidative, antiviral, antimicrobial, antiplatelet, and antitoxic activities [66]. The biological functions of these polyphenols in diverse biological systems are attributed to their redox characteristics, which are believed to play a pivotal role in the absorption and neutralization of free radicals, extinguishing singlet and triplet oxygens, or breaking down peroxides [67,68]. 

The antioxidant defense system in animals primarily relies on enzymes like CAT and SOD to protect cells from free radicals and facilitate their removal [28]. These enzymes serve as essential indicators of oxidative stress in aquatic animals [28,69]. In our study, we observed significantly higher levels of SOD and CAT activities in the liver, particularly in the group fed with *C. rotundus* (T3) at 0.8 g/kg. However, the activity decreased when a higher amount (1.6 g/kg) of *C. rotundus* was added. This decline may be attributed to the high sugar or carbohydrate content in the ethanolic *C. rotundus* extract. This, in turn, raises the possibility that this carbohydrate content contributes to the observed elevation in cholesterol levels. In line with our findings, a study on Nile tilapia [70] revealed the adverse effects of a high-cholesterol diet on liver function and mitochondrial abundance. This dietary regimen hindered endogenous cholesterol synthesis, upregulated genes associated with cholesterol esterification and efflux, and inhibited lipid catabolic processes, including mitochondrial β-oxidation and lysosome-mediated lipophagy, while also reducing insulin sensitivity [70].

Additional studies, such as one on juvenile turbot [71], have shown that high dietary cholesterol intake can affect key genes involved in cholesterol synthesis (*hmgcr*) and promote bile acid synthesis-related genes (*cyp7a1*). This was further corroborated by findings from studies on Japanese flounder (*Paralichthys olivaceus*) [72] and channel catfish (*Ictalurus punctatus*) [73], where increased body weight was associated with elevated plasma and hepatic cholesterol levels. Moreover, cholesterol accumulation in the liver, driven by high-carbohydrate diets, has been observed in Nile tilapia [74], potentially compromising hepatic antioxidant capacity [75]. Although high cholesterol intake seemed to stimulate growth in Nile tilapia, it also gave rise to metabolic disorders in the fish [70]. Our study supports these findings, as the T4 group exhibited high growth rates but showed signs of liver damage, indicated by elevated levels of serum MDA and AST. Serum AST levels are commonly employed as markers and indicators of illness in fish, providing insights into the extent of liver injury. These enzymes are released into the bloodstream, contributing to liver damage [76,77]. Furthermore, MDA serves as a marker for oxidative stress and damage to cellular membranes [69]. Typically, certain herbs, such as *Artemisia apiacea*, are known to exert antioxidant effects, resulting in significant increases in the activity of SOD and CAT in the livers of rats, accompanied by a marked reduction in MDA production [78]. Furthermore, the heightened activity of antioxidant enzymes has been observed to play a role in maintaining stable MDA levels in fish [79]. Based on these findings, it becomes apparent that a high dose of *C. rotundus* is associated with reduced antioxidant enzyme activity, leading to the elevation of AST and MDA levels. These observations suggest that *C. rotundus* not only contributes to an increase in cholesterol levels but may also be unsuitable for use in high concentrations as a component of tilapia feed. It is noteworthy that acute oral toxicity studies involving the administration of 95% ethanol-extracted *C. rotundus* rhizomes at a dosage of 5 g/kg and subacute toxicity studies entailing daily dosages of 1 g/kg over a 14 day period in rats did not yield toxic effects [80]. However, it is advisable to consider chronic toxicity evaluations to assess the long-term safety of the extract. Consequently, our research findings lead us to conclude that tilapia exposed to a high dose (1.6 g/kg of the diet) of *C. rotundus* over a 60 day period may experience chronic toxicity. The appropriate dose of *C. rotundus* for Nile tilapia was determined to be 0.8 g/kg of the diet, which proved suitable for promoting growth and maintaining blood parameters, as well as supporting optimal antioxidant enzyme activity. 

In previous studies of the immunology of Nile tilapia fed *C. rotundus*, it was demonstrated that *C. rotundus* aqueous extracts can be used for their antibacterial activity against *S. agalactiae*, as reported in the in vitro screening of candidate herbs [44]. Their results agree with our study showing *C. rotundus* enhancement of the non-specific immune system which appears to be the most promising approach for disease prevention in this fish. Following the administration of supplementation to the T3 group fed with *C. rotundus* (0.8 g/kg of the diet), a protective effect against *Streptococcus agalactiae* was observed. Upon incubation with fish serum, the bacterial counts showed a significant decrease, along with a notable improvement in the survival rate of Nile tilapia after being challenged with *S. agalactiae* for 14 days. This result may have been correlated with the elevation of nonspecific immune parameters such as LZM and MPO activity. After feeding, fish fed with *C. rotundus* showed enhancements in lysozyme activity in the T3 group. LZM is a crucial element within the immune system; it is present in tissues of various animals, including fish [81], and is utilized as a biomarker for evaluating the innate immune system’s ability to defend against microbial infections in the host [82,83]. 

Moreover, the bioactive constituents extracted from the tuber of *C. rotundus* exhibited significant antibacterial properties. Phenolic compounds, recognized as natural substances synthesized through secondary metabolites [84,85], play a crucial role in this context. Phenolic compounds possess diverse biological activities, including anti-inflammatory, antioxidant, and antimicrobial properties [86]. Notably, plant flavonoids exhibit antimicrobial effects, particularly targeting Gram-positive bacteria. The primary site of flavonoid action on Gram-positive bacteria is the cell membrane, potentially involving phospholipid bilayer damage, respiratory chain inhibition, or interference in ATP synthesis [87]. Tannins, another group of bioactive compounds, exert their antibacterial action by inhibiting fatty acid biosynthesis, demonstrating iron chelation activity, impeding iron uptake, and hindering cell wall synthesis. Additionally, tannins cause damage to the outer membrane and cell membrane [88]. Terpenoids present in essential oils also contribute to antibacterial activity and antioxidative potential, as evidenced by studies [89,90].

A singular instance within the previous literature highlights the effectiveness of *C. rotundus* extracts, not specifically in Nile tilapia but notably against the white spot syndrome virus (WSSV) in Indian white shrimp [43]. The present study constitutes the first documentation of the application of *C. rotundus* tuber extracts to enhance disease resistance in Nile tilapia. Our findings are consonant with previous studies that have reported an increase in disease resistance in Nile tilapia when exposed to various medicinal plants or their extracts [28,91,92,93,94]. Many studies suggest that medicinal plants possess immunomodulatory capabilities. The way a plant functions, either by enhancing the immune system or displaying anti-inflammatory effects, is mainly influenced by the particular plant species and its constituent active compounds. In certain instances, the combination of various bioactive compounds exhibits distinct behavior [61]. Thus, our results indicate that *C. rotundus*, a medicinal plant/herb, has immunomodulatory potential for use in Nile tilapia. 

## 5. Conclusions

This study investigated the feasibility of integrating the weed *C. rotundus* into fish feed. Our results indicate that adding *C. rotundus* at a level of 0.8 g/kg diet (T3) significantly improved immune responses and disease resistance in Nile tilapia. Furthermore, there was an observed upward trend in growth data; however, it was not statistically significant. These findings underscore the potential of *C. rotundus* as a natural product with promising applications in the aquafeed industry. The data presented herein offers valuable insights into the immunostimulant properties of *C. rotundus*, thereby emphasizing its potential significance in the context of tilapia culture practices.

## Figures and Tables

**Table 1:** Proximate analysis of experimental diets (% dry matter).

Chemical Composition	T1	T2	T3	T4
	(Control)	(0.4 g/kg Diet)	(0.8 g/kg Diet)	(1.6 g/kg Diet)
Crude lipid (%)	4.38	4.40	4.49	4.88
Crude fiber (%)	4.11	4.40	4.37	5.41
Crude protein (%)	36.48	35.20	35.17	34.18
Moisture (%)	12.92	15.07	14.67	14.49
Ash (%)	10.09	9.86	9.90	11.19
NFE% ^1^	44.94	46.14	46.06	44.34
GE (MJ/kg) ^2^	18.07	17.98	18.00	17.62

^1^ Nitrogen-free extract (NFE%) = 100 − (crude protein + crude lipid + ash + crude fiber). ^2^ Gross energy (GE) was calculated based on 23.6, 39.5 and 17.2 kJ/g protein, lipid, and carbohydrates, respectively.

**Table 2:** Preliminary phytochemical constituents of tuber *C. rotundus* ethanolic extract.

Phytochemicals	Results
Flavonoids	+
Alkaloids	-
Anthraquinones	-
Tannins	+
Saponins	-
Terpenoids	++
Steroids	-
Cardiac glycosides	-
Cyanogenic glycosides	-
Sugars/Carbohydrates	+++

+ present (+ = low; ++ = moderate; +++ high), - absent.

**Table 3:** Antioxidant activity and bioactive compounds of tuber *C. rotundus* ethanolic extract.

Antioxidant Activity and Bioactive Compounds	Extracts of *C. rotundus*
DPPH scavenging activity (mg TE/g of extract)	4.33 ± 0.04
FRAP activity (mg Fe^2+^/g of extract)	4.94 ± 0.05
TPC (mg GAE/g of extract)	7.98 ± 0.17
TFC (mg RE/g of extract)	9.15 ± 0.81

Data are given as mean ± SD (*n* = 3). 2,2-diphenyl-1-picrylhydrazyl assay (DPPH), Ferric reducing antioxidant power assay (FRAP), The total phenolic content (TPC), the total flavonoid content (TFC), GAE = gallic acid equivalent, RE = rutin equivalent.

**Table 4:** Growth performances and survival rate of Nile tilapia fed diets supplemented with *C. rotundus* ethanolic extract for 60 days.

Parameters	T1	T2	T3	T4
	(Control)	(0.4 g/kg Diet)	(0.8 g/kg Diet)	(1.6 g/kg Diet)
Weight gain (WG; g)	22.86 ± 1.93 ^b^	21.67 ± 4.67 ^b^	26.30 ± 0.80 ^ab^	28.60 ± 1.90 ^a^
Average daily growth gain (ADG; g/day)	0.38 ± 0.03 ^b^	0.36 ± 0.07 ^b^	0.44 ± 0.01 ^ab^	0.48 ± 0.03 ^a^
Specific growth rate (SGR; %/day)	1.94 ± 0.16	1.81 ± 0.36	1.96 ± 0.21	1.99 ± 0.17
Feed conversion ratio (FCR)	2.27 ± 0.26 ^ab^	2.59 ± 0.37 ^b^	1.90 ± 0.15 ^a^	2.16 ± 0.18 ^ab^
Survival rate (SR; %)	85.33 ± 6.11	82.66 ± 12.22	82.66 ± 6.11	84.00 ± 0.00

Data are given as mean ± SD. Different superscripts mean significant differences (*p* < 0.05).

**Table 5:** Blood chemical profiles of Nile tilapia fed diets supplemented with *C. rotundus* ethanolic extract for 60 days.

Parameters	T1	T2	T3	T4
	(Control)	(0.4 g/kg Diet)	(0.8 g/kg Diet)	(1.6 g/kg Diet)
Total protein (TP; g/dL)	3.86 ± 0.25	3.46 ± 0.30	3.86 ± 0.32	3.83 ± 0.05
Albumin (ALT; g/dL)	1.53 ± 0.11	1.16 ± 0.25	1.33 ± 0.30	1.36 ± 0.05
Globulin (GLB; g/dL)	2.33 ± 0.15	2.30 ± 0.09	2.53 ± 0.05	2.46 ± 0.11
Aspartate aminotransferase (AST; U/L)	66.00 ± 20.80 ^b^	64.00 ± 18.35 ^b^	60.33 ± 3.78 ^b^	98.33 ± 11.93 ^a^
Alanine aminotransferase (ALT; U/L)	25.33 ± 6.80	23.33 ± 9.07	24.33 ± 5.03	28.00 ± 3.46
Blood Urea Nitrogen (BUN; mg/dL)	2.14 ± 0.16	2.06 ± 0.12	2.13 ± 0.15	2.30 ± 0.20
Total cholesterol (TC; mg/dL)	121.33 ± 3.21 ^b^	123.00 ± 8.18 ^b^	128.00 ± 4.58 ^b^	144.01 ± 6.24 ^a^

Data are given as mean ± SD. Different superscripts mean significant differences in the rows (*p* < 0.05). No superscripts mean the values were not significantly different.

**Table 6:** Hematological values of Nile tilapia fed diets supplemented with *C. rotundus* ethanolic extract for 60 days.

Parameters	T1	T2	T3	T4
	(Control)	(0.4 g/kg Diet)	(0.8 g/kg Diet)	(1.6 g/kg Diet)
Red blood cell (RBC; ×10^6^ cells/mm^3^)	1.84 ± 0.07 ^ab^	1.98 ± 0.11 ^ab^	2.10 ± 0.13 ^a^	1.75 ± 0.06 ^b^
Hemoglobin (Hb; g/dL)	7.60 ± 0.60	8.06 ± 0.70	8.53 ± 0.70	7.40 ± 0.26
Hematocrit (Hct; %)	32.00 ± 3.46	34.66 ± 4.72	32.33 ± 2.08	31.66 ± 1.52
White blood cell (WBC; ×10^3^ cells/mm^3^)	2.20 ± 0.22	1.61 ± 0.44	1.50 ± 0.61	1.76 ± 0.29
Neutrophils (Neut; %)	42.33 ± 8.38	45.33 ± 8.62	43.33 ± 6.80	47.33 ± 10.59
Lymphocytes (Lymphs; %)	53.66 ± 9.29	52.67 ± 9.01	55.33 ± 6.65	50.67 ± 10.26
Mean cell volume (MCV; fL)	173.7 ± 9.07	175.70 ± 26.36	154.93 ± 22.44	180.43 ± 7.83
Mean cell hemoglobin (MCH; pg)	41.26 ± 2.12	40.66 ± 2.00	40.56 ± 1.80	42.10 ± 1.30
Mean corpuscular hemoglobin concentration (MCHC; g/dL)	23.83 ± 2.45	23.40 ± 2.60	26.40 ± 2.55	23.37 ± 1.09

Data are given as mean ± SD. Different superscripts mean significant differences (*p* < 0.05). No superscripts mean the values were not significantly different.

**Table 7:** The body composition (dry weight basis) and organosomatic indices of Nile tilapia fed diets supplemented with *C. rotundus* ethanolic extract for 60 days.

Parameters	T1	T2	T3	T4
	(Control)	(0.4 g/kg Diet)	(0.8 g/kg Diet)	(1.6 g/kg Diet)
Crude protein (%)	83.92 ± 0.54	83.55 ± 0.49	84.85 ± 0.94	85.11 ± 1.68
Crude lipid (%)	2.53 ± 0.09	2.87 ± 0.37	2.76 ± 0.08	2.54 ± 0.11
Moisture (%)	1.51 ± 0.01	1.53 ± 0.43	1.50 ± 0.02	1.36 ± 0.13
Ash (%)	6.19 ± 0.02	6.18 ± 0.50	6.20 ± 0.06	6.12 ± 0.11
Fillet (%)	25.42 ± 2.02 ^b^	24.54 ± 0.83 ^b^	26.10 ± 3.56 ^b^	29.66 ± 2.06 ^a^
Carcass (%)	57.47 ± 3.36	58.12 ± 1.76	56.07 ± 2.70	54.27 ± 1.86
Hepatosomatic index (HSI; %)	2.69 ± 0.54	3.10 ± 1.19	3.73 ± 0.71	3.53 ± 0.72
Viscerosomatic index (VSI; %)	10.26 ± 2.06	11.24 ± 1.51	10.93 ± 0.61	10.65 ± 1.47
Spleenosomatic index (SSI; %)	0.30 ± 0.08	0.28 ± 0.14	0.33 ± 0.10	0.26 ± 0.08

Data are given as mean ± SD. Different superscripts mean significant differences (*p* < 0.05). No superscripts mean the values were not significantly different.

**Table 8:** Immunological and antioxidant parameters of Nile tilapia fed diets supplemented with *C. rotundus* ethanolic extract for 60 days.

Parameters	T1	T2	T3	T4
	(Control)	(0.4 g/kg Diet)	(0.8 g/kg Diet)	(1.6 g/kg Diet)
Lysozyme activity	9.22 ± 4.34 ^b^	15.33 ± 11.77 ^ab^	29.55 ± 11.88 ^a^	21.27 ± 9.86 ^ab^
(LZM; U/mL)				
Myeloperoxidase	3.14 ± 0.45 ^a^	3.07 ± 0.36 ^a^	3.43 ± 0.32 ^a^	3.49 ± 0.12 ^a^
(MPO; OD_450nm_)				
Superoxide dismutase	13.12 ± 3.79 ^b^	15.93 ± 5.54 ^ab^	37.87 ± 4.09 ^a^	20.00 ± 9.53 ^ab^
(SOD; U/g liver)				
Malondialdehyde	94.82 ± 7.58 ^b^	87.25 ± 8.89 ^b^	99.16 ± 7.88 ^b^	117.39 ± 14.47 ^a^
(MDA; µmol/L)				
Catalase activity	37.50 ± 8.84 ^ab^	43.75 ± 8.84 ^a^	46.88 ± 8.07 ^a^	28.13 ± 8.07 ^b^
(CAT; U/g liver)				

Data are given as mean ± SD. Different superscripts mean significant differences (*p* < 0.05).

## Data Availability

The datasets generated during and/or analyzed during the current study are available from the corresponding author upon reasonable request.
